# Negative allosteric modulation of the mGlu7 receptor reduces visceral hypersensitivity in a stress-sensitive rat strain

**DOI:** 10.1016/j.ynstr.2015.04.001

**Published:** 2015-04-04

**Authors:** Rachel D. Moloney, Anna V. Golubeva, Richard M. O'Connor, Mikhail Kalinichev, Timothy G. Dinan, John F. Cryan

**Affiliations:** a Laboratory of Neurogastroenterology, Alimentary Pharmabiotic Centre, Biosciences Institute, University College Cork, Ireland; b Department of Psychiatry, University College Cork, Ireland; c Department of Anatomy and Neuroscience, University College Cork, Ireland; d Addex Therapeutics SA, Geneva, Switzerland

**Keywords:** Visceral pain, Anxiety, Wistar-Kyoto, Glutamate

## Abstract

Glutamate, the main excitatory neurotransmitter in the central nervous system, exerts its effect through ionotropic and metabotropic receptors. Of these, group III mGlu receptors (mGlu 4, 6, 7, 8) are among the least studied due to a lack of pharmacological tools. mGlu7 receptors, the most highly conserved isoform, are abundantly distributed in the brain, especially in regions, such as the amygdala, known to be crucial for the emotional processing of painful stimuli. Visceral hypersensitivity is a poorly understood phenomenon manifesting as an increased sensitivity to visceral stimuli. Glutamate has long been associated with somatic pain processing leading us to postulate that crossover may exist between these two modalities. Moreover, stress has been shown to exacerbate visceral pain. ADX71743 is a novel, centrally penetrant, negative allosteric modulator of mGlu7 receptors. Thus, we used this tool to explore the possible involvement of this receptor in the mediation of visceral pain in a stress-sensitive model of visceral hypersensitivity, namely the Wistar Kyoto (WKY) rat. ADX71743 reduced visceral hypersensitivity in the WKY rat as exhibited by increased visceral sensitivity threshold with concomitant reductions in total number of pain behaviours. Moreover, AD71743 increased total distance and distance travelled in the inner zone of the open field. These findings show, for what is to our knowledge, the first time, that mGlu7 receptor signalling plays a role in visceral pain processing. Thus, negative modulation of the mGlu7 receptor may be a plausible target for the amelioration of stress-induced visceral pain where there is a large unmet medical need.

## 1 Introduction

Glutamate signalling has long been implicated in the pathophysiology of pain states (Fundytus, 2001). Visceral pain is among the most poorly understood pain modality characterised by enhanced sensitivity to visceral stimuli. There is a dearth of information regarding the underlying mechanisms of visceral pain, however central sensitisation via excessive glutamatergic signalling has been implicated (Cervero, 2009, Cervero, 1995). Stress has been shown to be a critical factor in visceral pain pathophysiology, both in terms of increasing the risk to develop visceral hypersensitivity, and an exacerbating or perpetuating factor (Chaloner and Greenwood-Van Meerveld, 2013, Larauche et al., 2012, Johnson and Greenwood-Van Meerveld, 2014, Lee et al., 2015, Prusator and Greenwood-Van Meerveld, 2015, Hong et al., 2015, Theodorou, 2013, Jennings et al., 2014, Olango and Finn, 2014, Aguilera et al., 2013, Mayer et al., 2001b). Functional gastrointestinal disorders (FGIDs) such as irritable bowel syndrome (IBS) are typified by heightened visceral sensitivity as well as altered bowel movements and bloating. Stressful life events both early in life and in adulthood have long been implicated in the pathophysiology of IBS (Mayer, 2000, Mayer et al., 2001a, McEwen, 1998, Fukudo, 2013). Indeed numerous animal models used to investigate the pathogenesis of IBS are stress-based models of visceral hypersensitivity (Larauche et al., 2012, Moloney et al., 2015, Johnson and Greenwood-Van Meerveld, 2014). The potential role of glutamate in the nociceptive signalling of visceral stimuli has recently begun to be appreciated, both at a spinal and supra-spinal level (Lindstrom et al., 2008, Crock et al., 2012a, Blackshaw et al., 2011, Cao et al., 2014, Zhou et al., 2014).

Glutamate exerts its effects via ionotropic and metabotropic (mGlu) receptors (Golubeva et al., 2015). Eight mGlu receptors have been identified to date, which are sub-divided according to pharmacology, signal transduction pathways and sequence homology (Cartmell and Schoepp, 2000). mGlu receptors are not only expressed centrally but offer promising pharmacological targets for FGIDs as they are also expressed peripherally (Julio-Pieper et al., 2011). Furthermore, mGlu receptors are now being looked at more intensely as novel therapeutics not only for painful disorders but also psychiatric disorders due to the more attractive safety profile in comparison to their ionotropic counterparts which exhibit psychotomimetic side effects (Newcomer et al., 2000). Recent evidence supports the concept of targeting group 1 metabotropic receptors (mGlu1, mGlu5) as a potential therapeutic approach in animal models of visceral pain (Varty et al., 2005, Neugebauer and Carlton, 2002, Blackshaw et al., 2011, Crock et al., 2012b, Crock et al., 2012a). However, to date evidence for a role of group III receptors, specifically the mGlu7 receptor, is lacking.

From an evolutionary standpoint, mGlu7 receptors are the most highly conserved mGlu isoform (Flor et al., 1997). They are abundantly distributed in the brain, especially in those regions, such as the amygdala, known to be crucial for the emotional processing of painful stimuli (O'Connor et al., 2010). The mGlu7 receptor is of particular interest given that knockout and siRNA studies in mice have indicated altered amygdala-dependent conditioned fear and aversion responses (Callaerts-Vegh et al., 2006, Masugi et al., 1999, Fendt et al., 2008), and reduced anxiety- and stress-related behaviours (Cryan et al., 2003). Moreover, mGlu7 receptor ablation causes dysregulation of the hypothalamic–pituitary–adrenal (HPA) axis and increases hippocampal brain derived neurotrophic factor (BDNF) protein levels (Mitsukawa et al., 2006). More recently, the development of a novel negative allosteric modulator (NAM) of mGlu7 receptors, namely ADX71743 has been identified and characterised showing potential anxiolytic effects *in vivo* (Kalinichev et al., 2013). Taken together, these data may imply a potential role for the mGlu7 receptor in the modulation of visceral hypersensitivity which is comorbid with stress-related psychiatric disorders.

To this end, in the present study, we assessed whether ADX71743 ((+)-6-(2,4-dimethylphenyl)-2-ethyl-6,7-dihydrobenzo[*d*]oxazol-4(5*H*)-one), a potent, selective, and brain-penetrant mGlu7 NAM (Kalinichev et al., 2013) could reduce visceral hypersensitivity in the stress-sensitive Wistar Kyoto (WKY) rat strain. This strain has previously been shown to exhibit visceral hypersensitivity (Gunter et al., 2000, Carroll et al., 2013) in addition to increased anxiety-like behaviours (Hyland et al., 2015). In addition, previous *in situ* hybridization analysis from our lab has revealed that the WKY rats displayed selective increases in mGlu7 receptor mRNA expression in subregions of the hippocampus compared to Sprague Dawley controls (O'Mahony et al., 2010a). Thus, we also investigated whether negative modulation of mGlu7 could ameliorate the anxiogenic profile of this rat strain.

## 2 Materials and methods

### 2.1 Animals

Male WKY rats (250–300 g) (Harlan, UK) were used in this study. All animals were group housed in plastic cages (15 × 22 × 9 cm) and were maintained in a temperature controlled room (20 ± 1 °C) with a 12 h light/dark cycle. The animals were allowed one week to acclimatise to the animal facility in University College Cork after arrival. All experiments were conducted in accordance with the European Directive 2010/63/EU and approved by Animal Experimentation Ethics Committee of University College of Cork.

### 2.2 Experimental design

Two cohorts of animals were used in the current study.

a) **Cohort 1**: ADX71743 or vehicle was administered 30 min prior to colorectal distension (T0). Animals underwent the balloon insertion protocol 10 min later (T10) and allowed to recover until T30. Visceral pain behaviours were assessed at T30 and immediately after, the animals were euthanized.
b) **Cohort 2**: ADX71743 or vehicle was administered 30 min prior to the open field test (T0). 15 min after administration, a blood sample was taken (T15). The animals were introduced into the open field arena at T30. Animals were removed from the arena after 10 min at T40. Repeated blood samples were taken 5 min later (T45), 30 min later (T75) and 15 min later (T90).

### 2.3 Colorectal distension (CRD)

CRD was performed as previously described (O'Mahony et al., 2010b, O'Mahony et al., 2012). Briefly, animals were fasted overnight (16 h) and on the day of testing, were anaesthetised with isoflurane and a 6 cm latex balloon was inserted into the colorectal cavity, 1 cm from the anus. The animals were allowed to recover for 20 min before CRD commenced. The paradigm used was an ascending phasic distension from 0 mmHg to 80 mmHg over 8 min using a computer-driven electronic barostat (Dual Drive Barostat, Distender Series II, G & J Electronics Inc., Toronto, ON, Canada). The parameters of interest were: (1) the threshold pressure (mmHg) that evokes visually identifiable visceral pain behaviour and (2) the total number of pain behaviours. Postures defined as visceral pain behaviours were abdominal retractions and/or abdominal withdrawal reflex. The treatment groups were randomised and CRD was performed by an experimenter blinded to treatment groups.

### 2.4 Open field

Animals were allowed to habituate to the testing room 30 min prior to commencing the test. The open field apparatus consisted of a white round arena measuring 90 cm diameter, brightly lit to 1000 lux (Felice et al., 2014). Animals were introduced in to the centre of the arena one at a time and allowed to explore for 10 min. Animals were then removed and placed immediately back in their home cages. The arena was cleaned with 70% ethanol between each trial. Total distance travelled and the distance moved in the inner zone were analysed using a tracking software system (Ethovision, Noldus, The Netherlands). The treatment groups were randomised and the open field test was performed by an experimenter blinded to treatment groups.

### 2.5 Drug administration

ADX71743 was synthesised at Addex Therapeutics and kindly donated to us. ADX71743 (50, 100, 150 mg/kg) or vehicle (50% water solution of hydroxyl-propyl-β-cyclodextrin (CD)) were administered subcutaneously (s.c.) 30 min prior to commencement of behavioural testing (Kalinichev et al., 2013). The suspensions were homogenised with stainless steel balls for 30 min at 30 Hz in a 2-ml Eppendorf tube, and then vortexed and sonicated for 10 min. All drugs dosed s.c. were administered at 3 ml/kg volume. Suspensions were prepared fresh daily.

### 2.6 Blood sampling

To estimate the magnitude of plasma corticosterone (CORT) release upon exposure to a mild stressor, blood samples were collected before and after the open field protocol. Blood samples (200 μl each) were taken from the tail vein immediately prior to ADX71743 or vehicle administration (to determine baseline levels of CORT, T0), 15 min after the injection (T15), and at T45, T75 and T90 min after the open field test.

### 2.7 Corticosterone analysis

CORT levels in plasma were measured by ELISA (Enzo Life Sciences, Farmingdale, NY) according to the manufacturer's protocol.

### 2.8 Statistical analysis

All statistical tests were performed using SPSS 18. Graphing was performed using GraphPad Prism 5. All data was normally distributed according to Gaussian distribution analysis. Data are expressed as mean ± SEM. One-Way-Analysis of Variance (ANOVA) and LSD post hoc tests were used where appropriate. Stress-induced changes in CORT levels were analysed in mixed designed ANOVA with ADX71743 as a between-group factor and time as a repeated-measured within-group factor. Further between-group comparisons for each time point were done in one-way ANOVA followed by Tukey's post hoc tests. A p value <0.05 was deemed significant in all cases. N = 6–12/group.

## 3 Results

### 3.1 ADX71743 attenuates visceral hypersensitivity in the stress-sensitive Wistar Kyoto rat

**Fig. 1:**
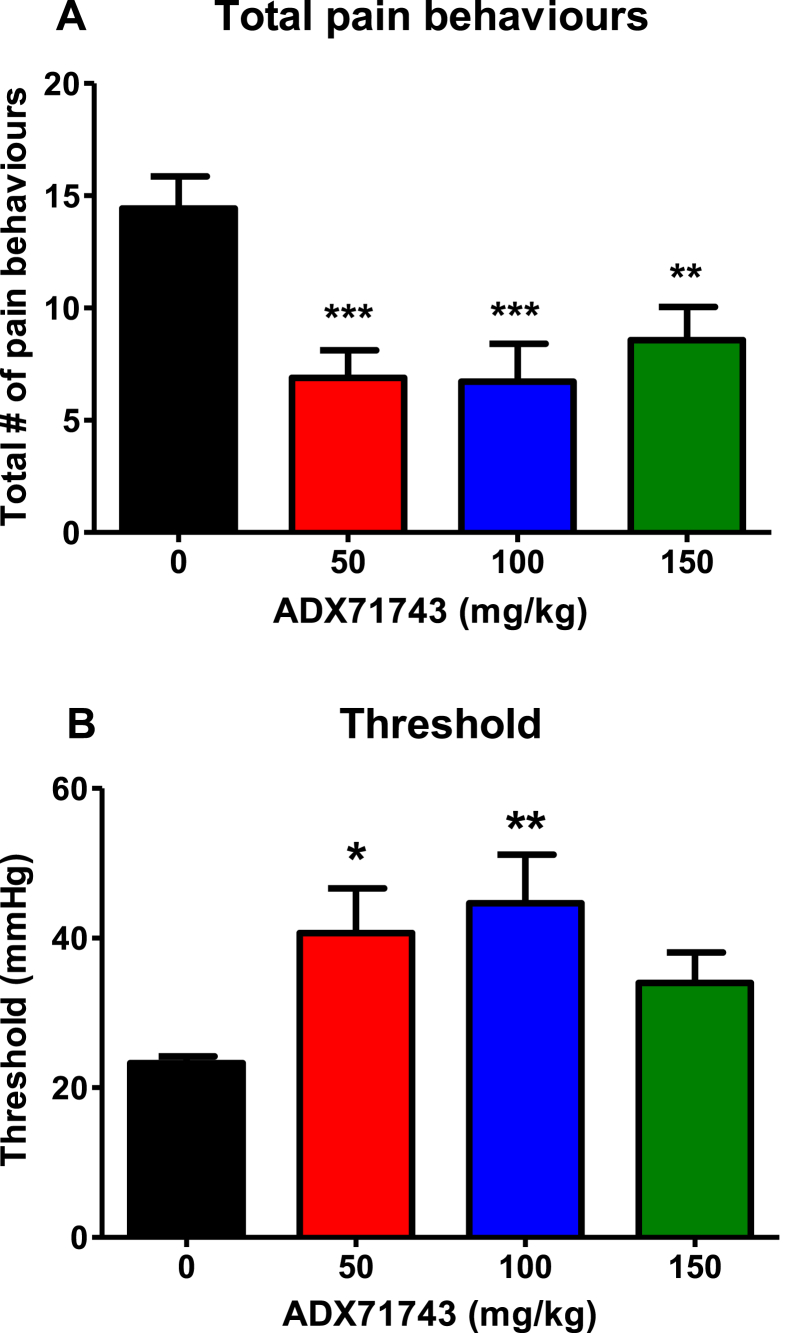
ADX71743 attenuates visceral hypersensitivity in the stress-sensitive Wistar Kyoto rat. Total pain behaviours [A] and sensitivity threshold [B]. (*p < 0.05, **p < 0.01, ***p < 0.001 vs vehicle, n = 12/group).

A one-way ANOVA analysis was performed to assess the effect of ADX71743 on visceral hypersensitivity, (both total pain behaviours and threshold). This revealed a significant effect of drug treatment for both total pain behaviours (F_(3,30)_ = 6.582, p < 0.01) and threshold (F_(3,30)_ = 3.092, p < 0.05). Post hoc analysis revealed that ADX71743 decreased total number of pain behaviours at all doses (Fig. 1A) and increased threshold sensitivity at the 50 mg/kg and 100 mg/kg doses (Fig. 1B) in the hypersensitive WKY rat (*p < 0.05, **p < 0.01, ***p < 0.001 vs vehicle).

### 3.2 ADX71743 attenuates anxiety-like behaviour in the stress-sensitive Wistar Kyoto rat

**Fig. 2:**
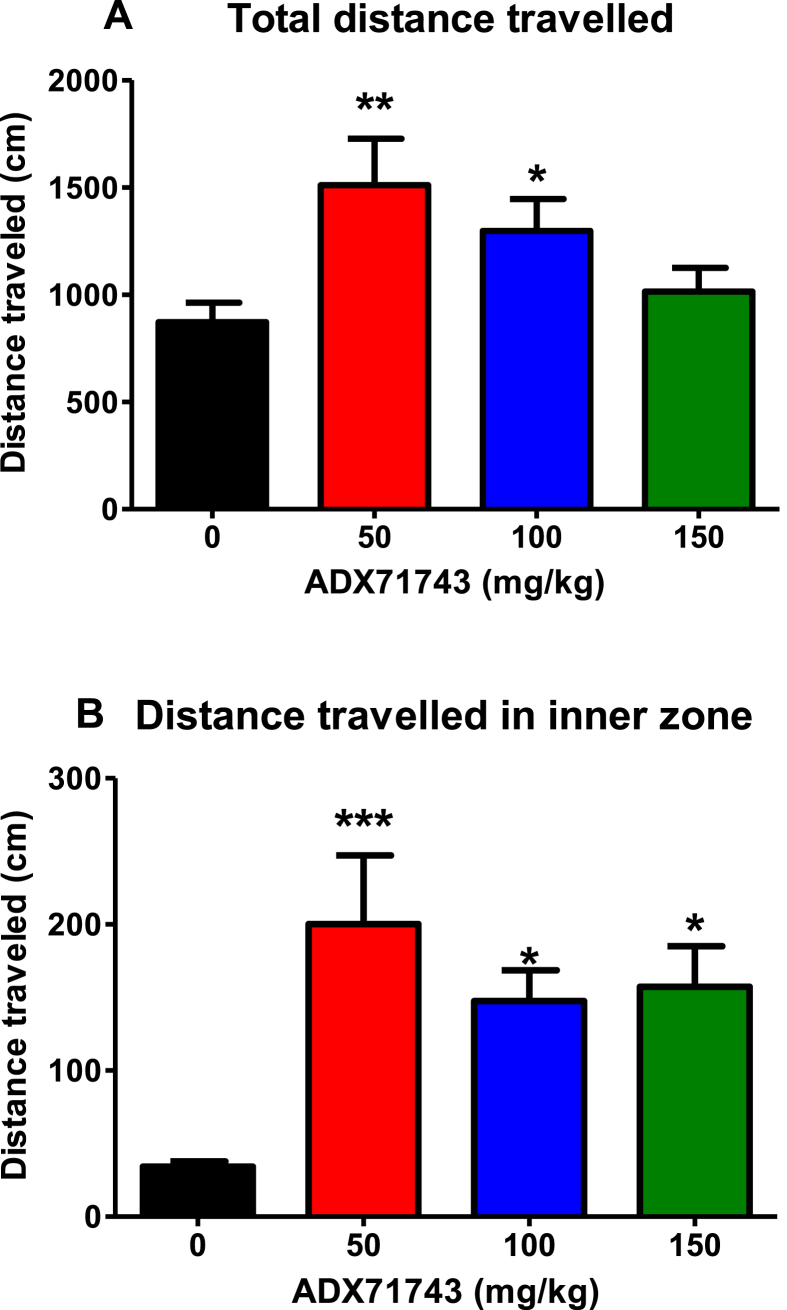
ADX71743 attenuates anxiety behaviour in the stress-sensitive Wistar Kyoto rat. Total distance travelled [A] and distance travelled in the inner zone [B]. (*p < 0.05, **p < 0.01, ***p < 0.001 vs control, n = 7–8/group).

A one-way ANOVA analysis was performed to assess the effect of ADX71743 on anxiety behaviour in the open field test, specifically total distance travelled and distance travelled in the inner zone. This revealed a significant effect of drug treatment for both total distance travelled (F_(3,30)_ = 5.936, p < 0.01) and distance travelled in the inner zone (F_(3,30)_ = 6.395, p < 0.01). Post hoc analysis revealed that ADX71743 increased the total distance travelled in the open field at doses of 50 mg/kg and 100 mg/kg (Fig. 2A). Moreover, ADX71743 dose-dependently increased the distance travelled in the inner zone of the open field at all doses (Fig. 2B), (*p < 0.05, **p < 0.01, ***p < 0.001 vs vehicle).

### 3.3 ADX71743 does not affect stress-induced corticosterone release in the stress-sensitive Wistar Kyoto rat

**Fig. 3:**
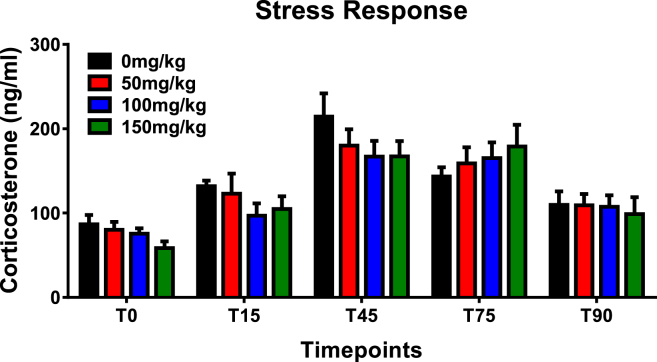
ADX71743 does not significantly alter the stress-induced corticosterone response in the stress-sensitive Wistar Kyoto rat (n = 6/group).

Baseline CORT levels were similar across all groups (p > 0.05, one-way ANOVA). Subcutaneous administration of either ADX71743 or vehicle induced an increase in plasma levels of CORT in average by 20–50 ng/ml (Fig. 3, T15). The subsequent exposure of animals to the novel arena stress under bright light resulted in a pronounced CORT spike in all groups (increased by 90–120 ng/ml), followed by a slow decline in CORT levels during the recovery period. A mixed-design ANOVA analysis was performed to assess the effect of ADX71743 on stress-induced corticosterone release. This revealed neither significant effect of drug treatment (F_(3,19)_ = 0.67, p = 0.581) nor significant drug × time interaction (F_(12,76)_ = 0.754, p = 0.694).

## 4 Discussion

We found that the mGlu7 negative modulator ADX71743 normalises visceral hypersensitivity in Wistar Kyoto rats as exhibited by reduced total visceral pain behaviours with a concomitant increase in the threshold to visceral sensitivity. Moreover, ADX71743 displays anxiolytic-like activity in the open field with enhanced total distance travelled and distance travelled in the inner zone. This is to our knowledge the first pharmacological study to implicate mGlu7 in visceral pain processes.

The group III mGlu receptors are not only the largest mGlu receptor group but have also been the least investigated due to a lack of selective pharmacological tools. The mGlu7 receptor is thought to be only activated during times of excessive glutamate release acting to inhibit further release. Indeed, excessive glutamatergic signalling has been shown to induce visceral pain behaviours (Cervero, 2000). The mode of action of mGlu7 receptors may lead us to postulate that their activation may be able to prevent the process of central sensitisation and thus exhibit analgesic properties. Indeed, treatment with mGlu7 receptor ligands such as mGlu7 receptor agonist, N,N(I)-dibenzhydrylethane-1,2-diamin dihydrochloride (AMN082) significantly inhibited both early and late phase formalin-induced hyperalgesia and pain behaviours (Dolan et al., 2011). Intrathecal injection of AMN082 post-carrageenan and post-surgery also significantly attenuated thermal hyperalgesia (Dolan et al., 2009). AMN082 has also been shown to decrease responses in a model of cardiac nociception (Liu et al., 2012). However, conflicting evidence has also emerged showing that AMN082 induces a hyperalgesic response in both the hotplate and tail flick assays (G et al., 2014)

However, although initially thought to be a useful tool to assess mGlu7 function (Mitsukawa et al., 2005, Fendt et al., 2008, O'Connor and Cryan, 2013) the finding that AMN082 has metabolites with strong monoamine activity (Sukoff Rizzo et al., 2011) detracts from it's overall utility. The current finding that negative modulation of mGlu7 receptors using ADX71743 ameliorates visceral pain is somewhat paradoxical as mGlu7 receptors act as auto-receptors to inhibit neurotransmitter release. By blocking mGlu7 receptors, one would assume that its auto-receptor function and thereby inhibitory effects on glutamate release would be prevented thus leading to excessive glutamate release into the synapse. However, similar findings have been reported by others using 6-(4-methoxyphenyl)-5-methyl-3-pyridin-4-ylisoxazolo[4,5-c]pyridin-4(5H)-one (MMPIP), an mGlu7 receptor negative allosteric modulator. It was found to block the first and second phase of nocifensive behaviour in the formalin pain model and increase tail flick latency (Palazzo et al., 2013).

Moreover, it is important to note that the effects seen in this study can be attributed to both central and peripheral sites of action. Further studies are required to fully elucidate the exact role of mGu7 receptors in the pathophysiology of visceral pain and to more accurately ascertain whether these analgesic effects are mediated peripherally or centrally. Indeed the complex pharmacodynamic properties of ADX71743 also require further investigation as eluded to in the 150 mg/kg treatment group, whereby its effects are less than that seen in the lower dose groups (50 and 100 mg/kg). This bell shaped curve suggests that lower doses of ADX71743 may prove more efficacious in the context of visceral pain and anxiety in the Wistar Kyoto model.

Similar questions arise when understanding why ADX71743 reduces anxiety behaviours in the open field test. Indeed comparable results have also been described by others, whereby ADX71743 was shown to decrease anxiety in the marble burying and elevated plus maze (EPM) tests (Kalinichev et al., 2013). Moreover, mGlu7 receptor knockout mice displayed anxiolytic-like behaviour in the light–dark box, EPM, marble burying test, the staircase test, and stress-induced hypothermia (SIH), as well as showing deficits in retention and extinction of conditioned fear (Callaerts-Vegh et al., 2006, Fendt et al., 2013, Cryan et al., 2003). Furthermore, mGlu7 receptor knock-down in adult brain using siRNA, reduced innate anxiety in the light–dark box, attenuated SIH response and fear-potentiated startle response (O'Connor et al., 2013). Moreover, ADX71743 has previously been evaluated in rodent models of psychosis again highlighting the regional localisation of mGlu7 within the mammalian CNS (Kalinichev et al., 2013). Although it is widely distributed throughout the brain, mGlu7 receptor shows particularly high abundance in the neocortex, piriform and entorhinal cortices, hippocampus, amygdala, globus pallidus, ventral pallidum, and the locus coeruleus (Kinoshita et al., 1998). Abnormalities in these regions have been linked to anxiety disorders (Walker and Davis, 2002), depression (Sanacora et al., 2012) and psychosis (Moghaddam and Adams, 1998) among other CNS disorders.

Recently, it was also postulated that the anxiolytic actions of ADX71743 may possibly involve the HPA axis (Kalinichev et al., 2013). This was based on findings from (Mitsukawa et al., 2006), showing that mGlu7 receptor knockout mice exhibit signs of HPA axis dysregulation, including up-regulation of glucocorticoid and 5-HT_1A_ receptors in the hippocampus, increased sensitivity to glucocorticoid-mediated negative feedback, and increases in BDNF protein in the hippocampus (Mitsukawa et al., 2006). These changes at a molecular level may indeed go towards explaining what is seen at a behavioural level with mGlu7 receptor KO animals exhibiting reduced anxiety- and depression-like behaviours (Cryan et al., 2003).

These findings suggest that inhibition of mGlu7 receptor signalling with ADX71743 would decrease the activity of the HPA axis. However, in the present study we did not observe a significant effect of ADX71743 on the HPA axis reactivity as estimated by similar magnitude of corticosterone release in response to an acute novelty stressor and the equal rate of post-stress recovery in the ADX71743-treated animals. These data somehow contradicts the results of previous study with subtype-nonselective group III receptor agonists (L-AP4, L-SOP) showing increased circulating levels of corticosterone in rats upon agonist treatment (Johnson et al., 2001). However, we should note that anxiolytic-like effect of different group III receptor modulators (such as AMN082, Lu AF21934, LSP1-2111, CPPG) has been shown to implicate different neurotransmitter systems (GABA, serotonin, or combination of both) (Stachowicz et al., 2007, Stachowicz et al., 2008, Slawinska et al., 2013, Wieronska et al., 2010). This can potentially contribute to the differential role of ADX71743 vs L-AP4/L-SOP in regulation of the HPA axis. Also, in the study by Johnson et al., Spraque–Dawley rats, an outbred strain, were used, which is believed to be more resilient to stress compared to the stress-sensitive WKY rat strain utilised in our study. Indeed our previous studies have shown that WKY animals have different physiological and behavioural response to early life and immune stressors (O'Mahony et al., 2013, Hyland et al., 2015, Felice et al., 2014). Genetically determined differences in the activity of stress-related neural circuits in these two strains could also underlie the observed discrepancies in the effects of ADX71743 and L-AP4/L-SOP on the HPA axis activity. Further studies utilizing various mGlu7 receptor ligands and different animal models are required to address the question whether inhibition of mGlu7 receptor can lead to alterations in the HPA axis.

Finally, we do not know whether the anti-nociceptive effects of ADX71743 are specific to stress-induced visceral pain and whether they generalise to other pain modalities. Future studies in this regard will also aid in unravelling the role of mGlu7 in pain processes. Furthermore, it is pertinent to note that in the current study the stress of drug administration via subcutaneous injection, had an impact on behavioural phenotypes in terms of both visceral sensitivity and anxiety-like behaviour.

Taken together, the data presented here add further evidence for the role of glutamate in the pathophysiology of visceral pain and moreover the use of ADX71743 as a promising new therapeutic for pain in functional bowel disorders with comorbid anxiety. However, the exact molecular mechanism by which ADX71743 mediates its effects must first be elucidated.

## Conflicts of interest

The authors state no conflict of interest.
